# Ion-acoustic waves dynamics in magnetized cometary plasma: nonlinear periodic and super-periodic waves with ion nonextensivity

**DOI:** 10.1038/s41598-025-89765-9

**Published:** 2025-03-04

**Authors:** S. Y. El-Monier, A. Atteya

**Affiliations:** 1 Arab Academy for Science & Technology and Maritime Transport, Alexandria, Egypt; 2 Department of Physics, Faculty of Science, Alexandria University, Alexandria, 21511 Egypt

**Keywords:** Comet plasma, Bifurcation, Solitons, Periodic waves, Superperiodic waves, Astronomy and planetary science, Physics

## Abstract

We explored the bifurcation analysis of ion-acoustic waves in a magnetized cometary plasma composed of hydrogen ions, positively and negatively charged oxygen ions, kappa-described hot solar electrons, and somewhat cooler cometary electrons. The modified Zakharov–Kuznetsov equation was derived using the reductive perturbation approach. Depending on the system’s characteristics, all conceivable phase pictures are provided, including periodic, homoclinic, and superperiodic trajectories. The presence of rarefactive and compressive solitary waves is demonstrated. Furthermore, the plasma system under discussion accommodates both nonlinear and supernonlinear periodic waves. It has been discovered that the nonextensivity of ions considerably alters the properties and has a considerable impact on the bifurcation of waves. The amplitudes of the solitary waves appear to be well connected with the existence of water molecules in cometary plasma, as well as the photoionization processes that accompany them.

## Introduction

The study of plasma systems spans various conditions and environments, from industrial applications to astrophysical phenomena. Plasma systems can be classified based on criteria such as temperature, density, magnetic field presence, ionization degree, and applications area. Recently, studying cometary plasma systems is crucial for understanding the formation and evolution of the Solar System, as these bodies preserve pristine materials from their early stages^1^. Recent research has provided fascinating insights into the complex composition and dynamics of these systems, revealing newborn heavier ions with densities that vary depending on their distance from the comet nucleus, along with hydrogen ions and electrons exhibiting different temperatures. Chaizy et al.^2^ discovered negatively charged oxygen ions within the coma of Comet Halley. This finding enabled the modeling of cometary plasma as a pair-ion plasma consisting of both $O^{+}$and $O^{-}$ ions, alongside other lighter and heavier ion species that collectively shape the ion composition. Moreover, various studies have confirmed multiple components of electrons within cometary environments, contributing to the overall complexity of these plasma systems^3–5^. Manesha et al.^6^ explore the influence of nonextensive ions on ion acoustic (IA) solitary waves in magnetized cometary plasmas. Through their theoretical analysis and numerical simulations, they examined how these nonextensive ions alter the nonlinear properties of ion-acoustic waves (IAWs), providing insights into the dynamics of cometary plasmas under varying conditions of ion distribution and magnetization. Nonlinear waves in plasma are studied using various equations and methods tailored to understand complex plasma dynamics^7–12^.

The Korteweg-de Vries (KdV) equation is fundamental for solitons and shallow water waves and has been adapted to describe IAWs in plasma systems^13^. The modified KdV (mKdV) equation includes higher-order nonlinear and dispersive effects, while the Zakharov-Kuznetsov (ZK) equation extends the KdV equation to higher-order nonlinearities and dispersion, particularly useful for envelope solitons in plasmas^14^. Further extensions include the extended ZK equation and modified ZK (mZK) equation, which accounts for additional nonlinear effects and were applied to study large-amplitude wave behaviors^15,16^. These equations are crucial for analyzing the propagation and behavior of nonlinear waves in plasma systems, contributing to advancements in plasma physics and astrophysical plasma dynamics. The IAWs are a significant type of nonlinear wave in plasma physics and were studied by many reasechers^7–12^. These waves are characterized by the oscillations of ions and electrons in response to perturbations in the electron density. They play a crucial role in various plasma environments, from laboratory experiments to natural and astrophysical plasmas. Sharma and Upadhyaya^16^ studied modified IA solitary waves in nonthermal plasmas, emphasizing the influence of non-Maxwellian distributions on wave characteristics. The Rosetta probe spotted IAWs between 15 and 30 kilometers from the center of comet 67P/Churyumov-Gerasimenko during its close approach on March 28, 2015. There are two electron populations: one cold at $k_{B} T_{e}\approx 0.2eV$ and another warm at $k_{B}T_{e}\approx 2eV$. The ions are dominated by a cool distribution of water group ions moving at bulk speed^17^. IA shock waves (shocklets) were investigated in a five-component cometary plasma using the Korteweg-deVries-Burger equation^18^, in which cometary electrons are characterized by kappa distribution functions with varying temperatures and spectral indices. Solitary waves were investigated in magnetized three-component quantum plasmas (electrons, positrons, ions) by deriving the ZK equation^19^. This revealed that soliton properties depend on Bohm potential, magnetic field strength, and thermodynamic parameters. The generation of IA shock waves in a magnetized, anisotropic plasma has been investigated in a five-component, cometary plasma^20^. They discovered that the magnetic field contributes to the parallel and perpendicular anisotropic ion pressures of positively and negatively charged oxygen ions.

Furthermore, the magnetic field has a significant impact on the propagation and breadth of shock waves, which have been referred to as “shocklets.” The width of these shocklets narrows as the magnetic field increases. EL-Monier et al.^21^ analyzed the fundamental characteristics and behaviors of nonlinear IA solitary and shock waves in magnetized multicomponent plasma. They found that several factors, such as components concentration, ion kinematic viscosity, magnetic field direction, and the superthermal parameter, influence the properties of oscillatory shock waves, including their speed, amplitude, and width.

Recently, in the context of dynamical system bifurcation analysis, some studies^22–36^ were published on exploring distinct qualitative qualities of diverse nonlinear wave patterns in various plasma systems. Using bifurcation analysis of dynamical systems, all conceivable phase plots, including superperiodic and superhomoclinic trajectories, are produced^31^. The dynamics of ions owing to the escape process due to solar wind induction and the escape of ions from the ionospheric shell of an unmagnetized planet, Venus, were researched. The modeling of IAWs was analyzed for the interaction between an upper ionospheric plasma of Venus. Additionally, the Sagdeev’s pseudopotential was investigated for wave-breaking events. All conceivable nonlinear and supernonlinear periodic waves were investigated using the phase plane analysis idea and the defined autonomous dynamical systems. The graph of the total energy function is displayed in three dimensions to support the presence of nonlinear and supernonlinear waves^33^. Bifurcations of nonlinear and supernonlinear IAWs were investigated in electron-ion plasmas with generalized (r, q) electron distributions. The ZK and mZK equations were used to evaluate the IAWs using the reductive perturbation approach. The translation of both the ZK and mZK equations into their respective dynamical systems allowed for the analysis of all feasible phase spaces and potential energy functions^34^. The dynamics of nonlinear and supernonlinear ion-acoustic waves were investigated in a four-component quantum plasma using the KdV and mKdV equations^35^. All conceivable phase portraits and accompanying small-amplitude Sagdeev’s pseudopotential for these dynamical systems were graphically shown. The phase portrait’s distinctive structure, as well as a peak between two minimas in pseudopotential curves, clearly indicate quantum IA superperiodic waves. Numerical methods were used to produce solitary, periodic, and superperiodic wave solutions in phase pictures, which correspond to homoclinic, periodic, and superperiodic orbits^35^. The wave solutions of the mKdV-Burgers equation were explored using planar dynamical system bifurcation theory in a four-component collisionless, unmagnetized plasma^36^.

The purpose of this work is to extend the previous work by Manesha et al.^6^ by investigating the 3-Dimension dynamics of IA solitary waves in a cometary plasma system, by applying the reductive perturbation approach to derive the mZK equation. The system under consideration comprises five constituents: negatively and positively charged oxygen ions, cometary (colder) and solar (hotter) kappa-characterized electrons, and hydrogen ions. The behavior of the heavier ions is governed by fluid equations, while the dynamics of the lighter ions ($H^{+}$) are controlled by a non-extensive distribution. This article is organized as follows: Section I displays the introduction. Section II contains the model equations, while Section III examines the MZK equation’s derivation and solution. Section IV is set for bifurcation analysis. Finally, the conclusions are represented in section V.

## Normalized-model equations

The IAWs are studied in magnetized, five-component cometary plasma composed of negatively and positively charged oxygen ions, hot electrons of solar origin, colder electrons of cometary origin, and hydrogen ions. At equilibrium, charge neutrality requires$\ Z_{-}n_{-0}-Z_{H}n_{H0}-Z_{+}n_{+0}+n_{ce0}+n_{se0}=0$, where $n_{-0}$, $n_{+0}$, $n_{H0}$ are the equilibrium densities of negatively charged oxygen ( $O^{-}$) ions, positively charged oxygen ($O^{+}$ ) ions, and hydrogen ($H^{+}$ ) ions, respectively. Also, $Z_{-}$, $Z_{+}$, and $Z_{H}$ denote their charge numbers, with $Z_{H}=1$. While $n_{ce0}$, $n_{se0}$ represent the equilibrium densities of cometary and solar electrons, respectively. The nonlinear dynamics of the IA waves for negatively and positively changed oxygen ions may be characterized as^6^

1 $$\begin{aligned} \left. \begin{array}{l} \frac{\partial n_{+}}{\partial t}+\nabla (n_{+}\textbf{u}_{+})=0,\\ \frac{\partial n_{-}}{\partial t}+\nabla (n_{-}\textbf{u}_{-})=0,\\ \frac{\partial u_{+}}{\partial t}+(u_{+}.\nabla )\textbf{u}_{+}=-\beta \nabla \phi +\omega _{+}\left( \textbf{u}_{+Y}\times i-\textbf{u}_{+x}\times j\right) ,\\ \frac{\partial u_{-}}{\partial t}+(\textbf{u}_{-}.\nabla )\textbf{u}_{-} =\nabla \phi -\omega _{-}\left( \textbf{u}_{-Y}\times i-\textbf{u}_{-x}\times j\right) ,\\ \nabla ^{2}\phi =n_{-}-n_{+}\left( 1+\delta _{ce}+\delta _{se}-\delta _{H}\right) -\left( 1+\delta _{ce}+\delta _{se}-\delta _{+}\right) n_{H}+\delta _{ce} n_{ce}+\delta _{se}n_{se}. \end{array} \right\} \end{aligned}$$

Here, $n_{-}$ and $n_{+}$ are the normalized densities of $O_{-}$ and $O_{+}$ ions, respectively, with $\textbf{u}_{-}$ and $\textbf{u}_{+}$ representing the corresponding normalized velocities and $\beta =\frac{Z_{+}m_{-}}{Z_{-}m_{+}}$. In Eq. (1), $\delta _{ce}=\frac{n_{ce0}}{Z_{-}n_{-0}},\delta _{se}=\frac{n_{se0}}{Z_{-}n_{-0}},\delta _{H}=\frac{n_{H} }{Z_{-}n_{-0}},$and $\delta _{+}=\frac{n_{+}}{Z_{-}n_{-0}}$ . The normalized number densities of the superthermal electrons encompassing both colder ($n_{ce}$), and hotter($n_{se}$) as well as the nonextensive hydrogen ions ($n_{H}$) are presented as

2 $$\begin{aligned} \left. \begin{array}{c} n_{ce}=\left[ 1-\frac{\phi }{\sigma _{ce}(k_{ce}-3/2)}\right] ^{-(k_{ce} -1/2)},\\ n_{se}=\left[ 1-\frac{\phi }{\sigma _{se}(k_{se}-3/2)}\right] ^{-(k_{s(e} -1/2)},\\ n_{H}=\left[ 1-\sigma _{H}(q-1)\phi \right] ^{\frac{3q-1}{2(q-1)}}, \end{array} \right\} \end{aligned}$$

where $\sigma _{ce}=\frac{T_{ce}}{T_{-}}$, $\sigma _{se}=\frac{T_{se}}{T_{-}}$, $\sigma _{H}=\frac{T_{-}}{T_{H}}$, and *q* is the nonextensive parameter

After substitution Eq. (2) in Eq. (1), the normalized Poisson’s equation will be given by

3 $$\begin{aligned} \nabla ^{2}\phi&=n_{-}-n_{+}\left( 1+\delta _{ce}+\delta _{se}-\delta _{H}\right) -\left( 1+\delta _{ce}+\delta _{se}-\delta _{+}\right) \left( 1-\sigma _{H}(q-1)\phi \right) ^{\frac{3q-1}{2(q-1)}}\nonumber \\&+\delta _{ce}\left[ 1-\frac{\phi }{\sigma _{ce}(k_{ce}-3/2)}\right] ^{-(k_{ce}-1/2)}+\delta _{se}\left[ 1-\frac{\phi }{\sigma _{se}(k_{se} -3/2)}\right] ^{-(k_{s(e}-1/2)}. \end{aligned}$$

## The mZK equation

To analyze the solitary wave characteristics in the magnetized plasma system under consideration, the mZK was derived by considering the reductive perturbation method is the used technique in which the employed stretched coordinates are represented as:

4 $$\begin{aligned} \left. \begin{array}{c} X=\epsilon x,Y=\epsilon y,Z=\epsilon (z-v_{0}t)\\ \tau =\epsilon ^{3}t, \end{array} \right\} \end{aligned}$$

where $\epsilon$ is a smallness parameter measuring the weakness of the dispersion ($0<\epsilon ~<1$). The coordinates stretched in Eq. (4) represent a Galilean transformation between two reference frames: (x, y, z, t) and (*X*, *Y*, *Z*, $\tau$). These frames are moving relative to each other at a constant speed $v_{0}$, which is assumed to be the phase velocity of the IA soliton. Through this transformation, it is evident that nonlinear structures in one frame can appear stationary in another, with perturbations dependent solely on spatial and temporal coordinates as defined by the combinations in Eq. (4). To derive the mZK equation using the reductive perturbation technique, the various parameters are expanded in a power series in $\epsilon$ as

5 $$\begin{aligned} \left. \begin{array}{c} n_{+}=\left( 1+\epsilon n_{+}^{\left( 1\right) }+\epsilon ^{2}n_{+}^{\left( 2\right) }+\epsilon ^{3}n_{+}^{\left( 3\right) }+...\right) ,\\ n_{-}=\left( 1+\epsilon n_{-}^{\left( 1\right) }+\epsilon ^{2}n_{-}^{\left( 2\right) }+\epsilon ^{3}n_{-}^{\left( 3\right) }+...\right) ,\\ u_{+x}=\left( \epsilon ^{2}u_{+x}^{\left( 1\right) }+\epsilon ^{3} u_{+x}^{\left( 2\right) }+\epsilon ^{4}u_{+x}^{\left( 3\right) }+...\right) ,\\ u_{+y}=\left( \epsilon ^{2}u_{+y}^{\left( 1\right) }+\epsilon ^{3} u_{+y}^{\left( 2\right) }+\epsilon ^{4}u_{+y}^{\left( 3\right) }+...\right) ,\\ u_{+z}=\left( \epsilon u_{+z}^{\left( 1\right) }+\epsilon ^{2} u_{+z}^{\left( 2\right) }+\epsilon ^{3}u_{+z}^{\left( 3\right) }+...\right) ,\\ u_{-z}=\left( \epsilon u_{-z}^{\left( 1\right) }+\epsilon ^{2} u_{-z}^{\left( 2\right) }+\epsilon ^{3}u_{-z}^{\left( 3\right) }+...\right) ,\\ \phi =\left( \epsilon \phi ^{\left( 1\right) }+\epsilon ^{2}\phi ^{\left( 2\right) }+\epsilon ^{3}\phi ^{\left( 3\right) }+...\right) . \end{array} \right\} \end{aligned}$$

After substituting Eqs. (4) and (5) in our system equations 1, and separating the lowest order terms of $\epsilon$ from the resultant equations, one can obtain the first order terms as follows for positive and negative ions

6 $$\begin{aligned} \left. \begin{array}{c} n_{+}^{\left( 1\right) }=\left( \frac{\beta }{V_{p}^{2}}\right) \phi ^{\left( 1\right) },u_{+z}^{\left( 1\right) }=\frac{\beta }{V_{p}} \phi ^{\left( 1\right) },\\ u_{+x}^{\left( 1\right) }=\left[ -\frac{\beta }{\omega _{c+}}\frac{\partial }{\partial Y}\phi ^{\left( 1\right) }\right] ,u_{+y}^{\left( 1\right) }=\left( \frac{\beta }{\omega _{c+}}\frac{\partial }{\partial X}\phi ^{\left( 1\right) }\right) ,\\ n_{-}^{\left( 1\right) }=\left[ -\left( \frac{1}{V_{p}}\right) ^{2} \phi ^{\left( 1\right) }\right] ,u_{-z}^{\left( 1\right) }=\left[ -\frac{1}{V_{p}}\phi ^{\left( 1\right) }\right] ,\\ u_{-x}^{\left( 1\right) }=-\frac{1}{\omega _{c-}}\frac{\partial }{\partial Y}\phi ^{\left( 1\right) },u_{-y}^{\left( 1\right) }=\frac{1}{\omega _{c-} }\frac{\partial }{\partial X}\phi ^{\left( 1\right) }. \end{array} \right\} \end{aligned}$$

**Fig. 1:**
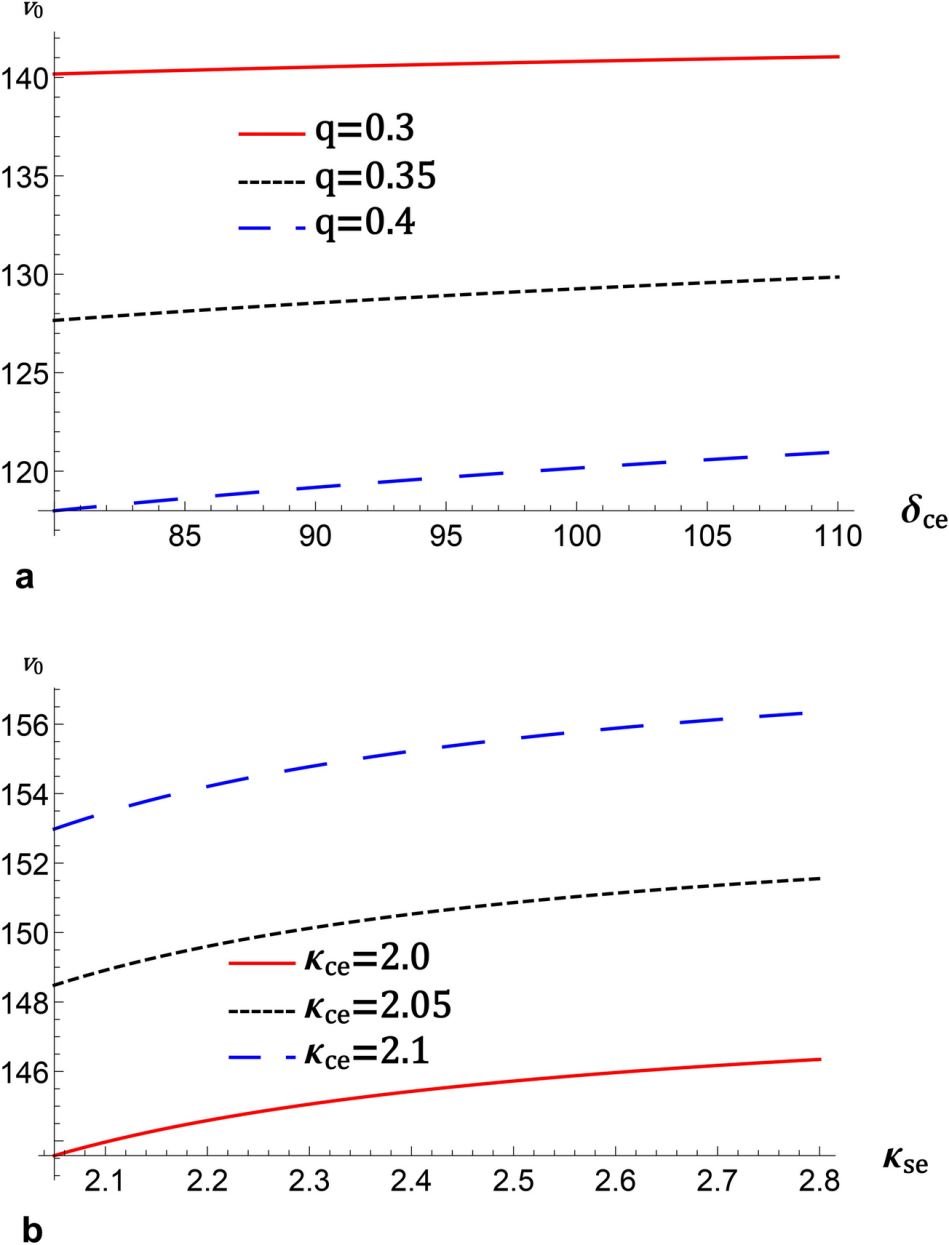
The variation of the phase velocity $v_{0}$, represented by Eq. 7 for $\sigma _{se}=20$, $\sigma _{ce}=2$, $\sigma _{H}=2$, and $\delta _{se}=90$ for (**a**) against cometary electrons to negative oxygen equilibrium densities ratio $\delta _{ce}$ for different values of nonextensive parameter, *q* at $k_{ce}=2$, and $k_{se}=2$, (**b**) against $k_{se}$ for different values $k_{ce}$ at $\delta _{ce}=90$, and $q=0.3$.

Now after substituting Eq. (6) in the Poission equation, the phase velocity of the waves will be obtained as follows

7 $$\begin{aligned} v_{0}=\sqrt{\frac{1+\beta \left( 1+\delta _{ce}+\delta _{se}-\delta _{H}\right) }{\gamma }}, \end{aligned}$$

with $\gamma =\frac{1}{2}(-1+3q)\left( 1+\delta _{ce}+\delta _{se}-\delta _{+}\right) \sigma _{H}+\frac{\delta _{ce}(-1+2k_{ce})}{(-3+2k_{ce})\sigma _{ce}}+\frac{\delta _{se}(-1+2k_{se})}{(-3+2k_{se})\sigma _{se}}$. The impact of slight fluctuation in nonextensive parameter *q* on the phase speed $v_{0}$ for distinct values of cometary electrons to-negative oxygen equilibrium densities ratio, $\delta _{ce}$ is presented in Fig. 1a. Also, the effects of superthermal parameters for both cometary (colder) and solar (hotter) electrons via $\kappa _{ce}$ and $\kappa _{se}$, respectively, on $v_{0}$ are also studied in Fig. 1b. it is depicted that, $v_{0}$ increases as $\delta _{ce}$, $\kappa _{ce}$ and $\kappa _{se}$ increase, while it decreases as *q* rises. This behavior can be attributed to the nonextensive effects introducing an additional dissipative term in the modified Korteweg-de Vries equation governing the IAWs propagation. This dissipative term depends on the perturbation in the nonextensive parameter *q*, leading to a reduction in the phase speed $v_{0}$^37^. Physically, the increase in the phase velocity due to the increase of cometary electron density, superthermal parameters of cometary and solar electrons, and can be attributed to the altered electron pressure and energy distribution, which significantly influence the ion dynamics and wave propagation in the plasma^38^.

By using the next order of $\epsilon$ in the Eq. (1), one can obtain the second-order perturbed quantities for Positive and negative ions as follows:

8 $$\begin{aligned} \left. \begin{array}{c} n_{+}^{\left( 2\right) }=\frac{3}{2}\frac{\beta ^{2}}{V_{p}^{4}} (\phi ^{\left( 1\right) })^{2}+\frac{\beta }{V_{p}^{2}}\phi ^{\left( 2\right) },\\ u_{+z}^{\left( 2\right) }=\frac{\beta }{V_{p}}\phi ^{\left( 2\right) } +\frac{1}{2}\frac{\beta ^{2}}{V_{p}^{3}}(\phi ^{\left( 1\right) })^{2},\\ n_{-}^{\left( 2\right) }=\left[ \frac{3}{2}\left( \frac{1}{V_{p}}\right) ^{4}\left( \phi ^{\left( 1\right) }\right) ^{2}-\left( \frac{1}{V_{p} }\right) ^{2}\phi ^{\left( 2\right) }\right] ,\\ u_{-z}^{\left( 2\right) }=\left[ -\frac{1}{V_{p}}\phi ^{\left( 2\right) }+\frac{1}{2V_{p}^{3}}\left[ \phi ^{\left( 1\right) }\right] ^{2}\right] . \end{array} \right\} \end{aligned}$$

By employing the next higher order of $\epsilon$ in the Eq. (1), the higher order of positive and negative ion density $n_{+}^{\left( 3\right) }$, and $n_{-}^{\left( 3\right) }$, respectively will be given by

9 $$\begin{aligned} & \frac{\partial n_{+}^{\left( 3\right) }}{\partial Z}=2\frac{\beta }{V_{p} ^{3}}\frac{\partial \phi ^{\left( 1\right) }}{\partial \tau }+3\frac{\beta ^{2} }{V_{p}^{4}}\frac{\partial \phi ^{\left( 1\right) }\phi ^{\left( 2\right) } }{\partial Z}+\frac{9}{4}\frac{\beta ^{3}}{V_{p}^{6}}\frac{\partial (\phi ^{\left( 1\right) })^{3}}{\partial Z}+\frac{\beta }{V_{p}^{2}} \frac{\partial \phi ^{\left( 3\right) }}{\partial Z}+\frac{\beta }{\omega _{c+}^{2}}\frac{\partial }{\partial Z}\left( \frac{\partial ^{2}\phi ^{\left( 1\right) }}{\partial X^{2}}+\frac{\partial ^{2}\phi ^{\left( 1\right) } }{\partial Y^{2}}\right) , \end{aligned}$$

10 $$\begin{aligned} & \frac{\partial n_{-}^{\left( 3\right) }}{\partial Z}=-\frac{2}{V_{p}^{3} }\frac{\partial \phi ^{\left( 1\right) }}{\partial \tau }+\frac{3}{V_{p}^{4} }\frac{\partial \phi ^{\left( 1\right) }\phi ^{\left( 2\right) }}{\partial Z}-\frac{9}{4V_{p}^{6}}\frac{\partial \left[ \phi ^{\left( 1\right) }\right] ^{3}}{\partial Z}-\frac{1}{V_{p}^{2}}\frac{\partial \phi ^{\left( 3\right) } }{\partial Z}-\frac{1}{\omega _{c-}^{2}}\frac{\partial }{\partial Z}\left( \frac{\partial ^{2}\phi ^{\left( 1\right) }}{\partial X^{2}}+\frac{\partial ^{2}\phi ^{\left( 1\right) }}{\partial Y^{2}}\right) . \end{aligned}$$

Finally, on substituting Eqs. (6)–(10) for these perturbed quantities in the third order of $\epsilon$ obtained from Poission’s equation, one can reach the mZK equation as

11 $$\begin{aligned} \frac{\partial \phi ^{\left( 1\right) }}{\partial \tau }+A\left( \phi ^{\left( 1\right) }\right) ^{2}\frac{\partial \phi ^{\left( 1\right) }}{\partial Z}+B\frac{\partial ^{3}\phi ^{\left( 1\right) }}{\partial Z^{3}} +C\frac{\partial }{\partial Z}(\frac{\partial ^{2}(\phi ^{\left( 1\right) } )}{\partial X^{2}}+\frac{\partial ^{2}(\phi ^{\left( 1\right) })}{\partial Y^{2}})=0, \end{aligned}$$

where *A* indeed corresponds to the nonlinear term, while *B* and *C* correspond to the longitudinal and transverse dispersive terms, respectively.

12 $$\begin{aligned} A&=\left( \frac{3V_{p}^{3}}{2(1+\beta \left( 1+\delta _{ce}+\delta _{se}-\delta _{H}\right) )}\right) \times \nonumber \\&\left( \begin{array}{c} \frac{9}{4V_{p}^{6}}-\frac{9}{4}\frac{\beta ^{3}}{V_{p}^{6}}\left( 1+\delta _{ce}+\delta _{se}-\delta _{H}\right) +\frac{1}{48}\left( 1+\delta _{ce}+\delta _{se}-\delta _{+}\right) \left( 3\sigma _{H}^{3} -7q\sigma _{H}^{3}-7q^{2}\sigma _{H}^{3}+3q^{3}\sigma _{H}^{3}\right) \\ +\delta _{ce}\frac{(-\frac{3}{2}-k_{ce})(-\frac{1}{2}-k_{ce})(\frac{1}{2}-k_{ce})}{6(-\frac{3}{2}+k_{ce})^{3}\sigma _{ce}^{3}}+\delta _{se} \frac{(-\frac{3}{2}-k_{se})(-\frac{1}{2}-k_{se})(\frac{1}{2}-k_{se})}{6(-\frac{3}{2}+k_{se})^{3}\sigma _{se}^{3}} \end{array} \right) ,\nonumber \\ B&=\frac{V_{p}^{3}\left[ \omega _{c-}^{2}\omega _{c+}^{2}+\omega _{c+} ^{2}+\omega _{c-}^{2}\beta \left( 1+\delta _{ce}+\delta _{se}-\delta _{H}\right) \right] }{2(1+\beta \left( 1+\delta _{ce}+\delta _{se}-\delta _{H}\right) )\omega _{c-}^{2}\omega _{c+}^{2}},\nonumber \\ C&=\left( \frac{V_{p}^{3}}{2(1+\beta \left( 1+\delta _{ce}+\delta _{se}-\delta _{H}\right) )}\right) . \end{aligned}$$

**Fig. 2:**
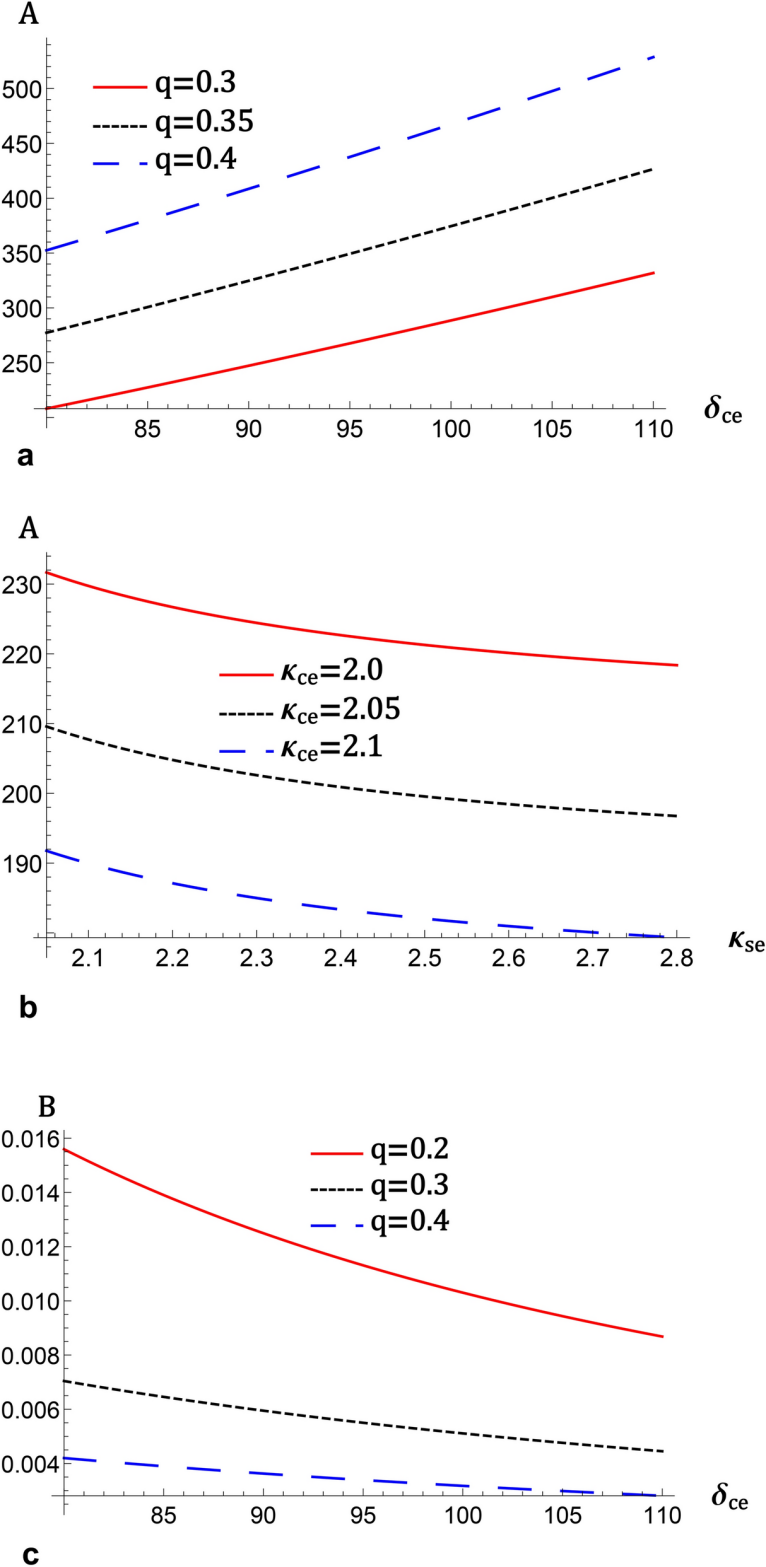

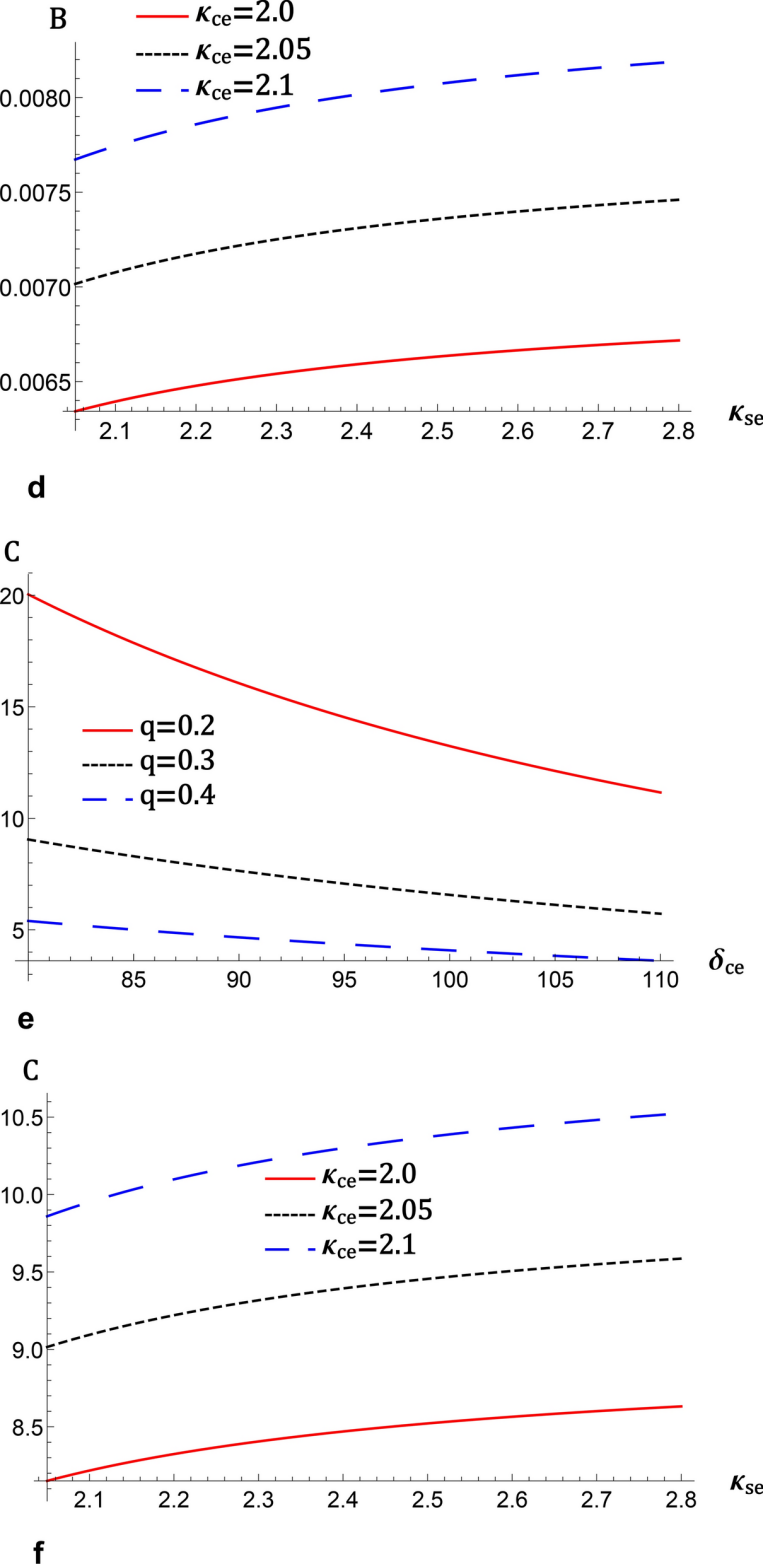
(**a**) The variation of the nonlinear term *A*, represented by (12) against $\delta _{ce}$ for different values of *q* at $k_{ce}=2$, and $k_{se}=2$ (**b**) the variation of *A* against $\kappa _{se}$ for different values $\kappa _{ce}$ at $\delta _{ce}=90$, and $q=0.3$, (**c**) the variation of the longitudinal dispersive term *B*, represented by (12) against $\delta _{ce}$ for different values of *q* at $\kappa _{ce}=2$, and $\kappa _{se}=2$, (**d**) the variation of *B* against $k_{se}$ for different values $k_{ce}$ at $\delta _{ce}=90$, and $q=0.3$, (**e**) the variation of the transverse dispersive term *C*, represented by (12) against $\delta _{ce}$ for different values of *q* at $k_{ce}=2$, and $k_{se}=2$, (**f**) the variation of *C* against $\kappa _{se}$ for different values $\kappa _{ce}$ at $\delta _{ce}=90$, and $q=0.3$, for $\sigma _{se}=20$, $\sigma _{ce}=2$, $\sigma _{H}=2$, $\delta _{se}=90$ , $\delta _{+}=10$, and $\omega _{c\mp }=0.5$.

These terms are affected by *q*, $\delta _{ce}$, $\kappa _{ce}$, and $\kappa _{se}$ as shown in Fig. 2. It is depicted that *A* increases (decreases) as *q* and $\delta _{se}$ ($\kappa _{ce}$ and $\kappa _{se}$) increase, while *B* and *C* decrease (increase) as *q* and $\delta _{se}$ ($\kappa _{ce}$ and $\kappa _{se}$) increase. This is due to the nonlinearity of IAWs increasing as superthermal parameters decrease because the distribution becomes more Maxwellian, leading to steeper wave structures. The increase in cometary electron density enhances the electrostatic potential and steepness of the waves, whereas the equilibrium density of these colder electrons increases, the plasma becomes more responsive to perturbations. This results in more pronounced density fluctuations and stronger nonlinear effects in the IAWs. Also, higher ion nonextensivity introduces more high-energy ions, amplifying nonlinear and reducing dispersion effects^21^.

## Bifurcation analysis

To investigate all travel wave solutions of Eq. (11), we transfer the mZK equation to the traveling wave system. We define the traveling coordinate $\chi$ as $\chi =\left( l_{x}X+l_{y}Y+l_{z}Z-\lambda \tau \right)$, where $l_{x}$, $l_{y}$, and $l_{z}$ are the directional cosines of the wave propagation vector, $\textbf{k}$, concerning the *X*, *Y*, and *Z* axes, respectively. The velocity of the moving frame, $\lambda$, is normalized by the ion’s acoustic speed. Substituting $\phi ^{(1)}\left( \chi \right) =$ $\phi ^{(1)}\left( X,Y,Z,\tau \right)$ into Eq. (11) yields the mZK equation:

13 $$\begin{aligned} -\lambda \frac{\partial \phi ^{\left( 1\right) }}{\partial \chi }+Al_{z}\left( \phi ^{\left( 1\right) }\right) ^{2}\frac{\partial \phi ^{\left( 1\right) } }{\partial \chi }+\left[ Bl_{z}^{3}+C\left( l_{z}-l_{z}^{3}\right) \right] \frac{\partial ^{3}\phi ^{\left( 1\right) }}{\partial \chi ^{3}}=0. \end{aligned}$$

Integrate Eq. (13) concerning $\chi$ and neglect the integration constant.

14 $$\begin{aligned} -\lambda \phi ^{(1)}+\frac{1}{3}Al_{z}\left( \phi ^{(1)}\right) ^{3}+\left[ Bl_{z}^{3}+C\left( l_{z}-l_{z}^{3}\right) \right] \frac{\partial ^{2} \phi ^{\left( 1\right) }}{\partial \chi ^{2}}=0. \end{aligned}$$

Therefore, Eq. (14) takes the following form

15 $$\begin{aligned} \frac{\partial ^{2}\phi ^{\left( 1\right) }}{\partial \chi ^{2}}=a\phi ^{(1)}-b\left( \phi ^{(1)}\right) ^{3}, \end{aligned}$$

with

$$\begin{aligned} a=\frac{\lambda }{\left[ Bl_{z}^{3}+C\left( l_{z}-l_{z}^{3}\right) \right] } \& \text b=\frac{A}{3\left[ Bl_{z}^{2}+C\left( 1-l_{z}^{2}\right) \right] }. \end{aligned}$$

Equation 15$\times \partial \phi ^{(1)}$ and by integration w.r.t $\phi ^{\left( 1\right) }$

16 $$\begin{aligned} \frac{1}{2}\left( \frac{\partial \phi ^{\left( 1\right) }}{\partial \chi }\right) ^{2}=\frac{a}{2}\left( \phi ^{(1)}\right) ^{2}-\frac{b}{4}\left( \phi ^{(1)}\right) ^{4}. \end{aligned}$$

The following dynamical system can be obtained as a result of Eq. (15)

17 $$\begin{aligned} \left. \begin{array}{c} \frac{d}{d\chi }\phi ^{(1)}=z,\\ \frac{d}{d\chi }z=\left( a-b\left( \phi ^{(1)}\right) ^{2}\right) \phi ^{(1)}. \end{array} \right\} \end{aligned}$$

The system (17) is a planar Hamiltonian system with a Hamiltonian function:

18 $$\begin{aligned} H\left( \phi ^{\left( 1\right) },\frac{\partial \phi ^{\left( 1\right) } }{\partial \chi }\right) =\frac{1}{2}\left( \frac{\partial \phi ^{\left( 1\right) }}{\partial \chi }\right) ^{2}+\frac{b}{4}\left( \phi ^{(1)}\right) ^{4}-\frac{a}{2}\left( \phi ^{(1)}\right) ^{2}=Energy. \end{aligned}$$

The system (17) is a dynamical system, with parameters $\delta _{se}$, $\delta _{ce}$, $\sigma _{se}$, $\sigma _{ce}$, $k_{ce}$, $k_{se}$ and $\lambda$. It is worth noting that the phase orbits given by the vector fields of 15 are responsible for all mZK traveling wave solutions. We study the bifurcations of phase pictures of (17) in the ($\phi ^{(1)}$, *z*) phase plane when the parameters $\delta _{se}$, $\delta _{ce}$, $\sigma _{se}$, $\sigma _{ce}$, $k_{ce}$, $k_{se}$ and $\lambda$ change. In this scenario, we investigate a physical system for which only bounded traveling wave solutions are relevant. As a result, we need to focus on the mZK equation’s bounded traveling wave solutions. The mZK’s solitary wave solution corresponds to a homoclinic orbit of (17). This work relies heavily on the bifurcation theory of planar dynamical systems^39,40^.

For the system (17), we obtain the fixed three equilibrium points at $E_{1}(0,0)$, $E_{2}\left[ \left( \frac{a}{b}\right) ^{1/2},0\right]$ and $E_{3}\left[ -\left( \frac{a}{b}\right) ^{1/2},0\right]$ resulted from the bifurcations of the phase portraits of Eq. (17) in the phase plane of ($\phi ^{(1)},d\phi ^{(1)}/d\chi$). The coefficient matrix of the linearized system (Jacobian matrix) is $$\left( \begin{array}{cc} 0 & 1\\ a & 0 \end{array} \right)$$ at $E_{1}(0,0)$, the Jacobian matrix value becomes $J_{E_{1} (0,0)}=-a$, but the Jacobian matrix at $E_{2}\left[ \left( \frac{a}{b}\right) ^{1/2},0\right]$ and $E_{3}\left[ -\left( \frac{a}{b}\right) ^{1/2},0\right]$ is $$\left( \begin{array}{cc} 0 & 1\\ -2a & 0 \end{array} \right)$$ and $$\left( \begin{array}{cc} 0 & 1\\ -2a & 0 \end{array} \right)$$it takes the value $J_{E_{2}\left[ \left( \frac{a}{b}\right) ^{1/2},0\right] }=J_{E_{3}\left[ -\left( \frac{a}{b}\right) ^{1/2} ,0\right] }=2a$.

The Hamiltonian system’s equilibrium point is a saddle point if $J<0$, and a center if $J>0$. When $J=0$, the equilibrium point’s Poincare index equals zero, indicating that the point is a cusp^39^.

Now, considering a potential energy function $V\left( \phi ^{\left( 1\right) }\right)$ for the system (17), we have

19 $$\begin{aligned} \frac{1}{2}\left( \frac{\partial \phi ^{\left( 1\right) }}{\partial \chi }\right) ^{2}+V\left( \phi ^{\left( 1\right) }\right) =0, \end{aligned}$$

where the Sagdeev potential $V\left( \phi ^{\left( 1\right) }\right)$ according to Eq. (16) is given by

20 $$\begin{aligned} V\left( \phi ^{\left( 1\right) }\right) =\frac{b}{4}\left( \phi ^{(1)}\right) ^{4}-\frac{a}{2}\left( \phi ^{(1)}\right) ^{2}. \end{aligned}$$

**Fig. 3:**
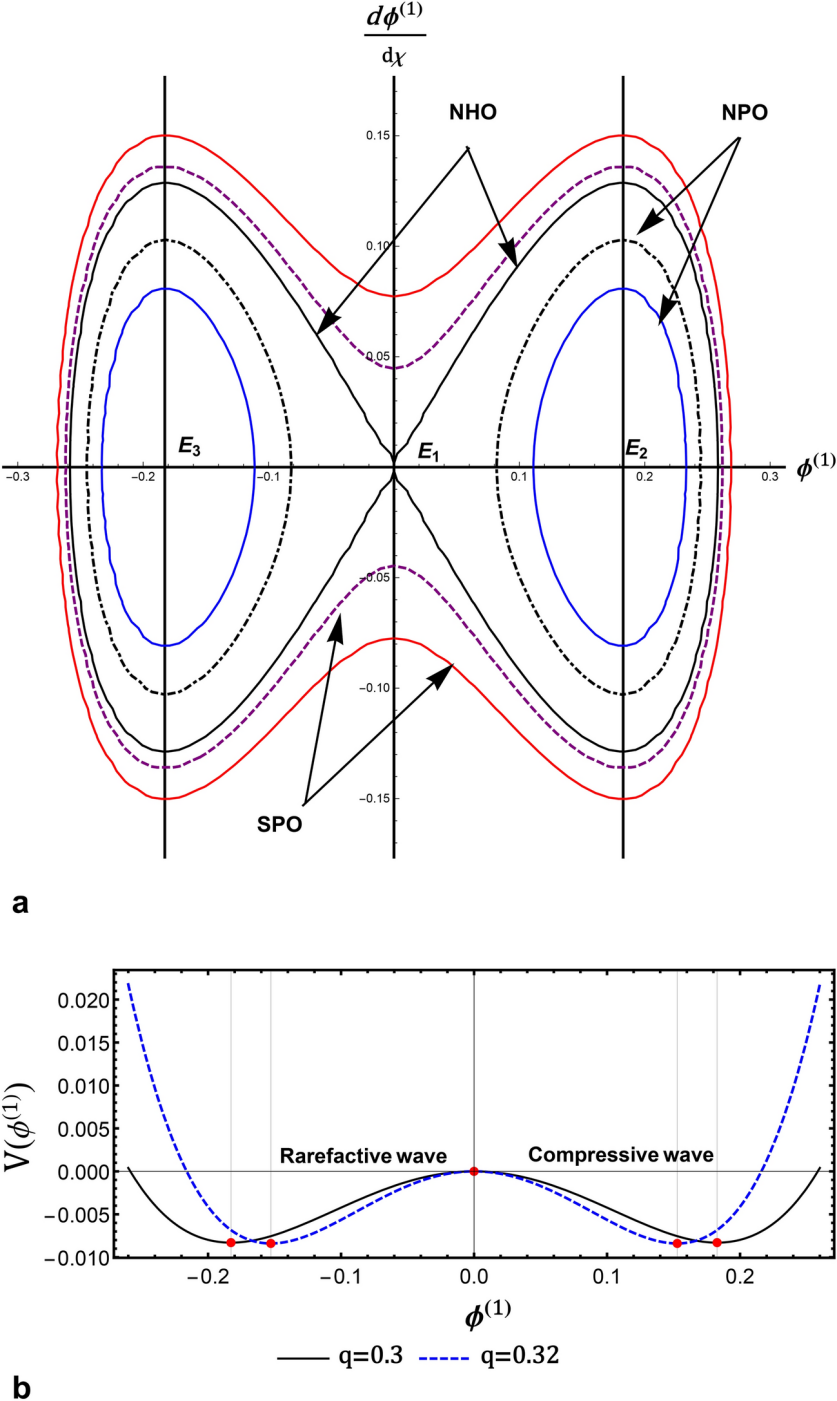
(**a**) Phase plots of system (17) for $\lambda =0.8$, $q=0.3$, $\sigma _{H}=2.8$, $\delta _{ce}=90$, $\delta _{se}=110$, $\sigma _{se}=20$, $\sigma _{ce}=2$, $\kappa _{ce}=2$, $\kappa _{se}=2$ , $\delta _{+}=10$, $\beta =16$, and $\omega _{c\mp }=0.5$, (**b**) Sagdeev potential $$V\left( \phi ^{\left( 1\right) }\right) \ $$against the potential $\phi ^{\left( 1\right) }$ for two different values of *q* with the same parameters as in a.

In Fig. 3, we show (a) the phase portrait profile and (b) the potential energy function for the dynamical system (17) using the mZK Eq. (11). The parameter settings for this study are based on the work^6^. In Eq. (17), the nonlinear periodic orbit (NPO) corresponds to a nonlinear periodic IAWs solution, while the nonlinear homoclinic orbit (NHO) corresponds to a nonlinear IA solitary wave solution. We found NPO and NHO that enclose points $E_{1}$ and $E_{3}$ on both positive and negative areas of the phase plane. Here, we witness the presence of a novel kind of wave called a supernonlinear periodic wave associated with supernonlinear periodic orbit (SPO) which formed around the fixed points $E_{1}$, $E_{2}$, and $E_{3}$ with one separatrix.

The negative and positive areas correspond to rarefactive and compressive solitary wave solutions, also known as IAW solitons. Figure 3b depicts the potential energy function for the relevant dynamical system (17), which includes two local minima and one maximum with superperiodic waves, compressive, and rarefactive solitons. The influence of *q* on Sagdeev pseudopotential is displayed in Fig. 3b. This figure shows that the depth of Sagdeev potential is diminishing with the decrease in *q*, leading to a decrease in the steepness of the solitary wave. It is shown that the wave width broadens with increasing *q*, indicating an amplification of the amplitude of the compressive solitary wave.

Keeping the remaining parameters of Fig. 3 constant, we see a small-amplitude supernonlinear periodic wave corresponding to the dynamical system (17) of the mZK equation (11) for $q>0.1$. This study is in good accord with the parametric range provided in the paper^33–35,41^ for the analysis of slow solar wind streams. Thus, we may assume that the superperiodic properties of IAWs under the mZK equation (11) is a unique result discovered in the cometary plasma and the associated photoionization processes.

**Fig. 4:**
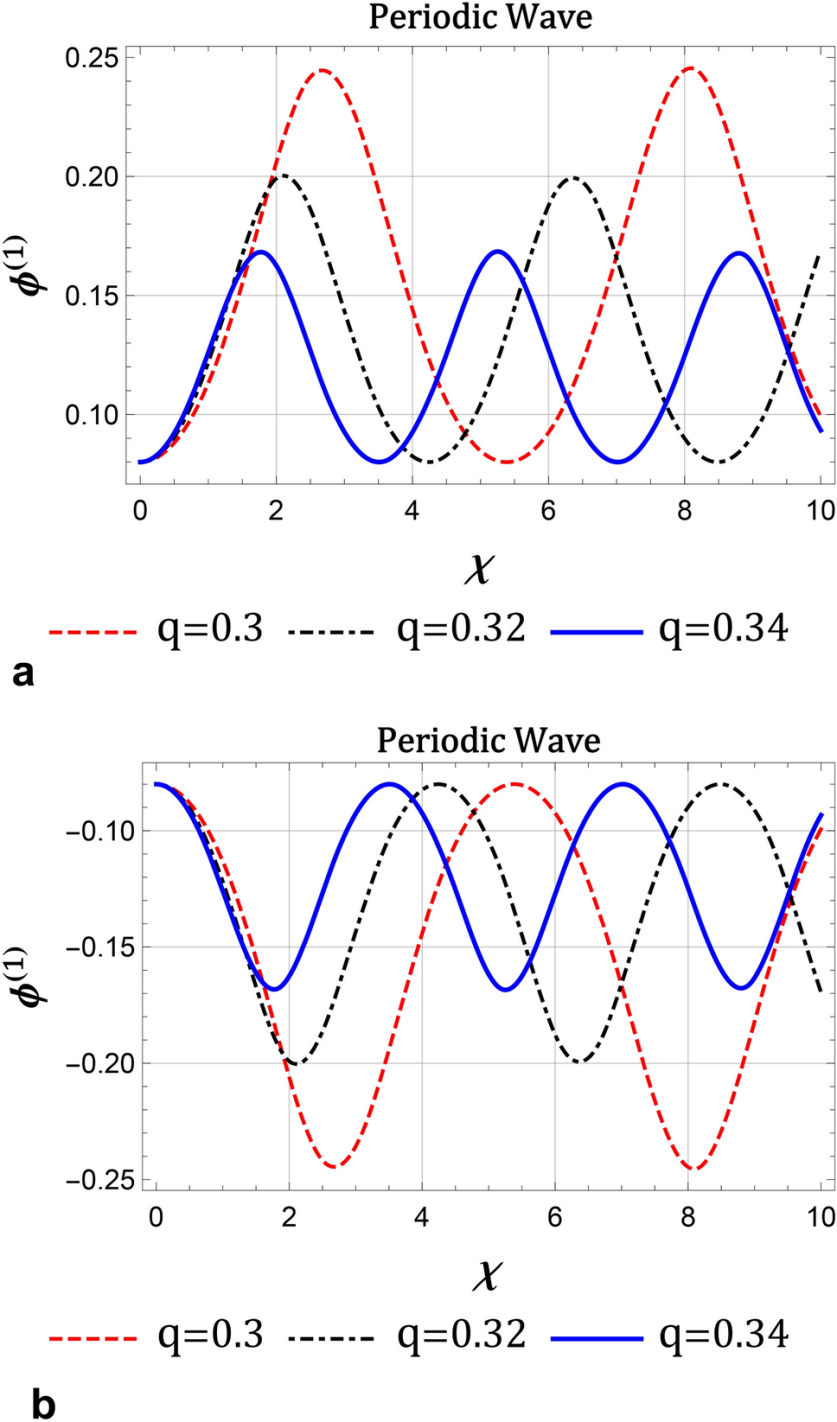
The evolution of $\phi ^{(1)}$ for different values of *q* (**a**) for compressive IA periodic waves (**b**) for rarefactive IA periodic waves with the same values of other parameters as in figure 3.

**Fig. 5:**
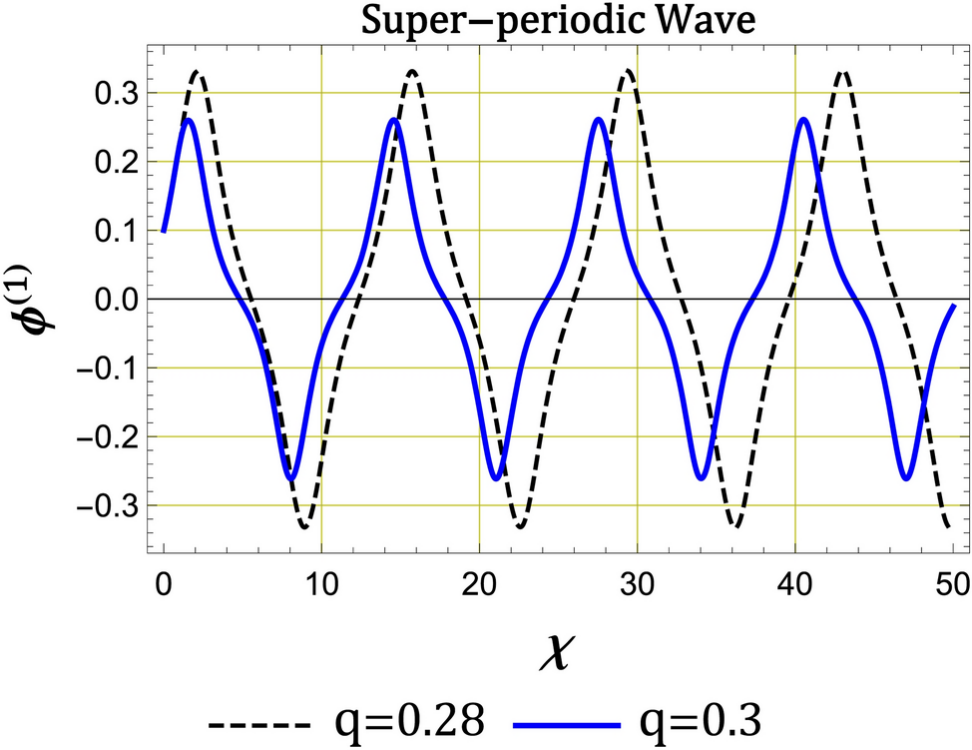
The evolution of $\phi ^{(1)}$ for different values of *q* for IA super-periodic waves with the same values of other parameters as in figure 3.

**Fig. 6:**
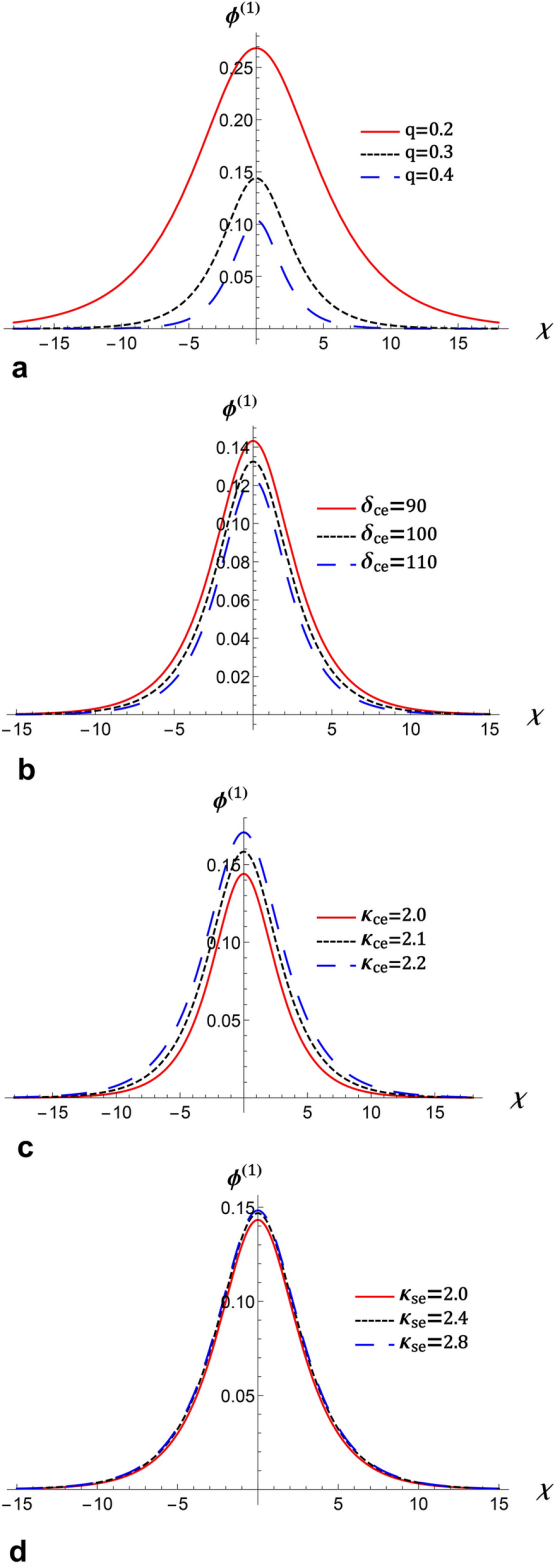
The evolution of $\phi ^{(1)}$ of IA solitary waves that represented by Eq. (21) against $\chi$ for different values of (**a**) *q* at $\kappa _{ce}=2$, $\kappa _{se}=2$, and $\delta _{ce}=90$, (**b**) $\delta _{ce}$ at$\ q=0.3$, $\kappa _{ce}=2$, and $\kappa _{se}=2$, (**c**) $\kappa _{ce}$ at $\delta _{ce}=90$, $q=0.3$, and $\kappa _{se}=2$, and (**d**) $\kappa _{se}$ at$\ \kappa _{ce}=2$, $q=0.3$, and $\delta _{ce}=90$ with the same values of other parameters as in Fig. 3.

Numerically, we present nonlinear periodic waves corresponding to NPO under the mZK equation (11) in Fig. 4 with the parameter values fixed as Fig. 3. Here, we observe from Fig. 4 that when *q* is increased, amplitudes of compressive and rarefactive periodic waves decrease, also the widths are reduced. We also present IA supernonlinear periodic waves (IASPWs) corresponding to supernonlinear periodic orbits under the mZK equation (11) in Fig. 5. This figure presents small-amplitude IASPWs with parameter values fixed as Fig. 3. Here, we observe that the width of IASPWs is highly influenced by changes in *q*. As *q* increases, width of IASPWs decreases while the amplitude increases as observed in Fig. 4. The homoclinic orbit at (0, 0) refers to a solitary wave profile. Also, since the parameters $\lambda$ and *B* are positive, the stable solitonic solution is given by^34^

21 $$\begin{aligned} \phi ^{(1)}=\phi _{m}\operatorname {sech}\left[ \frac{\chi }{\Delta }\right] , \end{aligned}$$

where $\phi _{m}=\sqrt{6\lambda /A}$ is the amplitude and $\Delta _{m} =\sqrt{B/\lambda }$ is the soliton width. Figure 6 presents the solitary wave solution associated with NHO. Here, we have compressive IASWs solutions corresponding to positive regions of orbits presented in Fig. 3. Here, Fig. 6a–d shows that when the *q* and $\delta _{ce}$ are increased, the amplitude and the width of IASW decrease, where the waves become smooth. Also, when the $\kappa _{ce}$ and $\kappa _{se}$ are increased, the amplitude and the width of IASW increase, or we can say, the waves exhibit increased complexity.

## Conclusions

This study provides a detailed examination of IAWs in a magnetized cometary plasma, focusing on the impact of ion nonextensivity. The plasma model includes hydrogen ions, positively and negatively charged oxygen ions, kappa-distributed hot solar electrons, and cooler cometary electrons. By deriving the mZK equation using the reductive perturbation approach, we have revealed the complex dynamics of IAWs, which exhibit periodic, homoclinic, and super-periodic trajectories.

A key finding is the significant influence of ion nonextensivity on wave characteristics. The nonextensive parameter *q* plays a crucial role in altering wave amplitudes, stability, and bifurcation behavior. Specifically, variations in *q* affect the steepness and width of solitary waves, highlighting the importance of ion distribution and energy levels in shaping wave dynamics. Additionally, the presence of water molecules and associated photoionization processes in the cometary plasma are closely linked to the characteristics of these waves. The study also underscores the importance of electron temperature differences, with cooler (0.2 eV) and hotter (2.0 eV) electron populations influencing wave stability and propagation. The results of this study should be useful to understand the characteristics of IA periodic and superperiodic waves in astrophysical and space plasma containing non-Maxwellian ions and kappa-described hot electrons of solar origin and colder electrons of cometary origin, which reflect the mixing of solar wind plasma and newly ionized cometary plasma, which is crucial for understanding the fundamental plasma processes, energy transfer, and large-scale structures that develop as the comet interacts with the solar wind. The study of superperiodic waves can also provide insights into cometary properties, such as the activity level of the comet, the structure of the diamagnetic cavity, and the role of waves in shaping the plasma environment.

## Data Availability

The datasets used and/or analyzed during the current study are available from the corresponding author upon reasonable request.
